# Non-communicable Disease Risk Factor Profile Among the Fishing Community in Ernakulam District of Kerala: A Community-Based Cross-Sectional Study

**DOI:** 10.7759/cureus.80644

**Published:** 2025-03-16

**Authors:** Kavya K R, Jaideep C Menon, Niveditha Kartha, Brilly M Rose

**Affiliations:** 1 Department of Community Medicine, Amrita Institute of Medical Sciences and Research Centre, Kochi, IND; 2 Department of Cardiology, Amrita Institute of Medical Sciences and Research Centre, Kochi, IND; 3 Department of Biostatistics, Amrita Institute of Medical Sciences and Research Centre, Kochi, IND

**Keywords:** behavioral risk factors, fishing community, hypertension, kerala, lifestyle factors, non-communicable diseases, obesity, public health, risk factors

## Abstract

Background

Non-communicable diseases (NCDs) significantly contribute to the global disease burden, with fishing communities being particularly vulnerable due to occupational and lifestyle factors. This study assesses the NCD risk factor profile among the fishing community in the Ernakulam district of Kerala, India.

Methods

A cross-sectional study was conducted among 415 participants, including fishermen and fisherwomen aged 18 years and older. Data were collected using a standardized questionnaire for NCD risk factor assessment, and a multistage cluster sampling method was used. Statistical analysis was performed using IBM SPSS version 20.0 (IBM Corp., Armonk, NY, USA). The chi-square test was used to assess associations between behavioral and sociodemographic risk factors with NCDs. Multivariable logistic regression was employed to identify significant predictors of NCDs.

Results

The mean age of participants was 51.3 ± 4.99 years, with 72.3% (300) men and 27.7% (115) women. Key behavioral risk factors included current tobacco use (28.7%), past tobacco use (58.1%), current alcohol use (56.6%), inadequate fruit and vegetable intake (88.9%), and inadequate sleep (90.1%). Hypertension was present in 57.8% of participants, overweight/obesity in 62.2%, and abdominal obesity in 93%. Significant predictors of hypertension included advancing age, past smoking, and inadequate sleep. Overweight/obesity was significantly higher among men, and abdominal obesity was strongly associated with past smoking.

Conclusion

The fishing community exhibited a high prevalence of NCD risk factors, emphasizing the urgent need for targeted interventions. Addressing behavioral risk factors, improving dietary habits, and promoting healthy physical activity and adequate sleep are crucial to reducing NCD risks. An integrated, community-based approach is recommended to enhance health outcomes in this marginalized population.

## Introduction

Non-communicable diseases (NCDs) have become a significant global health concern, contributing to over half of the global disease burden in recent decades [1]. According to the World Health Organization (WHO), annual mortality from NCDs is projected to rise from 38 million in 2012 to 55 million by 2030 [2]. In 2020, NCDs accounted for 71% of global deaths (41 million), underscoring their epidemic proportions [3].

India, with a population of 1.3 billion, accounts for more than two-thirds of all NCD-related deaths in the WHO South-East Asia Region, with nearly 5.9 million deaths annually attributed to conditions such as cardiovascular diseases, chronic respiratory disorders, cancer, and diabetes [4,5]. Alarmingly, one in four Indians faces the risk of dying from an NCD before the age of 70 [6,7].

Kerala, a southern state in India, reports a significantly higher prevalence of NCDs compared to national averages. The state has notable rates of diabetes (21.9%) vs. 8.9% nationally [8,9], cardiovascular diseases (19.9%) [10], and chronic respiratory disorders (10%) vs. 2.7% nationally, highlighting the urgent need for targeted NCD prevention and management strategies [11,12].

The fishing community, integral to Kerala's economy, faces unique occupational and lifestyle risks that contribute to a heightened NCD burden. Fishermen are exposed to hazardous working conditions, including prolonged hours at sea, unsanitary environments, and limited access to healthcare [13]. Fisherwomen, heavily involved in fishery-related activities, are similarly vulnerable to health risks. These occupational and environmental exposures necessitate focused research to understand the determinants of NCDs within this underserved community [14].

Despite the evident risks, research addressing the health needs of fishing communities, particularly in Kerala, remains scarce. Most studies to date have concentrated on fishermen, often neglecting fisherwomen and other subgroups within these communities [15]. Given the distinct risk profile of this population, tailored approaches to NCD prevention are essential. This study aims to assess the prevalence and determinants of NCDs in Kerala’s fishing population while also exploring their awareness of NCDs and treatment-seeking behaviors. Findings will inform the development of targeted preventive and health promotion strategies for this high-risk group.

## Materials and methods

A cross-sectional study was conducted in Njarakkal Panchayat, Ernakulam district, Kerala. Ernakulam has 21 fishing villages, and Njarakkal is one among them, with a total of 518 households [16]. This study was conducted in Njarakkal due to its significant fishing population and feasibility within resource constraints. Future studies may consider a multi-village approach for broader generalizability.

The study population consisted of the fishing community residing in Njarackkal Panchayat. For the purpose of this study, the fishing community was defined as "individuals who are significantly engaged in or reliant on the harvesting or processing of fishery resources to fulfill their social and economic needs, including fishing vessel owners, operators, crew members, and fish processors." Participants included both fishermen and fisherwomen aged 18 years or older. Individuals who did not provide consent or had not been actively engaged in the fishing profession for more than six years were excluded from the study. This six-year threshold ensures participants are actively engaged in fishing-related occupations and have recent occupational exposures, improving the relevance and accuracy of the study findings.

Sample size estimation

The sample size was calculated based on a previous study on fishermen from another district of Kerala that reported the prevalence of NCD risk factors such as tobacco use (43%), alcohol consumption (26.5%), dietary habits (36.5%), physical inactivity (10.4%), overweight (27.9%), abdominal obesity (30%), and hypertension (36.2%). Following statistical recommendations, the least prevalence among these risk factors was chosen to ensure an adequately powered sample size. As a result, the prevalence of alcohol consumption (26.5%) was used for sample size calculation [17]. The sample size was estimated using a single proportion formula. Since cluster sampling was used, the sample size was adjusted with a design effect of 1.5, resulting in a final sample size of 415 participants, selected using the probability proportional to size method to ensure adequate representation from different clusters within the study area. A non-response rate was not considered in the sample size estimation because engagement levels were expected to be high in this well-defined community, and data collection involved direct face-to-face interviews, minimizing non-response. Additionally, probability proportional to size sampling ensured adequate representation, and practical constraints limited the feasibility of further adjustments.

Sampling method

A multi-stage cluster sampling technique was used to select study participants. Njarakkal Panchayath was purposively selected as the study area from the Ernakulam District, Kerala, due to its high concentration of the fishing community and its relevance to the study objectives. The Panchayath consists of 16 wards, each considered a cluster. Since there were variations in the population sizes across these wards, all 16 clusters were included in the study to ensure comprehensive representation. To achieve proportional representation, the probability proportional to size (PPS) method was used to determine the number of participants selected from each ward. Within each selected household, one eligible participant was chosen randomly using a lottery method.

Data collection

Permission to survey the target population was obtained from the Njarakkal Panchayath president and chairman of the Standing Committee for Health. A detailed geographical map of Njarakkal Panchayath was obtained with the help of medical social workers from the Amrita rural health center in Njarrackkal. After obtaining written informed consent from the participants, a face-to-face interview was carried out by a trained investigator. Data was collected using the WHO STEPS Questionnaire for NCD risk factor assessment (STEP 1 & STEP 2), Version 3.0 (Appendix 1) [18]. The questionnaire included steps 1 and 2. STEP 1 (questionnaire) was to gather data on socio-demographic parameters and behavioral risk factors such as tobacco use, alcohol use, dietary habits, sleep duration, and physical activity. STEP 2 (physical measurements) was used to collect physical measurements such as blood pressure, Body Mass Index (BMI) (height and weight), waist circumference, and pulse rate. The investigator received training in standardized measurement techniques from doctors in the Community Medicine Department at Amrita Institute of Medical Sciences, Kochi, Kerala. Pilot testing and immediate verification of responses were implemented to maintain data quality. The total study period was from December 2020 to May 2021 (six months).

Study variables

Hypertension, overweight and obese, and abdominal obesity were our dependent variables. Hypertension was defined based on the Eighth Joint National Committee (JNC 8) classification of hypertension, according to which participants are said to be hypertensive if they have stage 1 hypertension (HTN) (systolic blood pressure (BP): 140-159 mmHg; diastolic BP: 90-99 mmHg) or stage 2 HTN (systolic BP: >=160 mmHg; diastolic BP: ≥100 mmHg). Blood pressure was measured using a calibrated digital sphygmomanometer following standard guidelines. Participants rested for five minutes before measurement, and two readings were taken, with a third if needed, averaging the last two. Measurements were done in private settings using appropriate cuff sizes. A trained investigator ensured accuracy, and participants with elevated readings were informed and referred for care. Data were securely recorded for analysis. Overweight and obesity were defined based on the WHO BMI classification, according to which overweight and obese were defined as those who had a BMI ≥ 25 kg/m2. Abdominal obesity was classified based on the Asian cut-off for waist circumference. A waist circumference of ≥ 90 cm in men and ≥ 80 cm in women was considered abdominal obesity.

Socio-demographic risk factors such as age, gender, level of education, type of occupation, duration in occupation, number of hours spent in occupation, and monthly income, and behavioral risk factors such as tobacco use, alcohol use, improper diet, physical inactivity, and inadequate sleep are the independent study variables. Tobacco use was defined based on two categories: current tobacco users and past tobacco users. Current tobacco users were defined as those who were using any type of tobacco products, such as cigarettes, cigars, beedis, or chewed tobacco, for the past year. Past tobacco users were defined as those who quit usage of any type of tobacco product for more than one year and have not used it since. Alcohol users were classified into current alcohol users and past alcohol users. Current alcohol users were defined as those who had used alcoholic beverages over the past year. Past alcohol users were defined as those who had quit all forms of alcohol consumption for more than one year. Adequate fruit and vegetable intake is defined based on servings of fruits and vegetables. One serving was considered to be 80 g of fruits and vegetables. The consumption of five or more servings of fruits and vegetables was considered to be adequate fruit and vegetable intake. Individuals consuming less than five servings of fruits and vegetables were considered to have an inadequate fruit and vegetable intake. Data on physical activities was collected using the Global Physical Activity Questionnaire (GPAQ), developed by the World Health Organization to assess the physical activity performed by a person in one week (Appendix 1)[19]. As our study population, the fishing community engages in moderate to vigorous physical activities as a part of their profession. Physical inactivity in our study is defined as those who are performing less than 75 minutes of vigorous-intensity physical activity per week. Inadequate sleep was defined based on recommended hours of sleep per day by the Centers for Disease Control and Prevention (CDC), according to which those who have a sleep duration of less than six hours a day are considered to have inadequate sleep [20].

Statistical methods

Statistical analysis was performed using SPSS statistics (version 20.0, IBM Corp., Armonk, NY, USA). Categorical variables were expressed using frequency and percentage. To test the statistical significance of the difference in the proportion of behavioral risk factors and socio-demographic risk factors with hypertension, overweight and obese, and abdominal obesity, the chi-square test was applied. After univariate analysis, variables with a p-value of <0.2 were included in a backward stepwise logistic regression model. A p-value of <0.05 was considered statistically significant.

Ethical consideration

The clearance from the institutional ethical committee was obtained before the commencement of the study from the Amrita Institute of Medical Sciences, Kochi. Informed consents were obtained from the study participants. Privacy and confidentiality of the participants and the data collected were assured.

## Results

The study included 415 participants, with a majority of 60.5% (251) aged between 41 and 60 years, while 14.2% (59) were in the 18-40 years category and 25.3% (105) were above 60 years. Gender distribution showed that 72.3% (300) of the participants were male, while 27.7% (115) were female. A majority, i.e., 65.5% (272), had completed primary school education or below, whereas only 34.5% (143) had attained at least secondary education. The most common occupation was fishing with 65.5% (272), followed by fish peeling with 17.3% (72), fish vending with 11.1% (46), and fishnet repair with 6% (25). The socioeconomic classification, based on the modified Kuppuswamy scale, indicated that the majority, i.e., 57.3% (238), belonged to the lower class, followed by 40.7% (169) in the upper lower class, and only 1.9% (8) in the lower middle class. 45.5% (189) of the participants worked 21-40 hours per week, while 42.9% (178) worked 0-20 hours, and only 11.6% (48) exceeded 40 hours per week. Additionally, most participants, i.e., 93.7% (389), reported working for one to 14 continuous hours, whereas only 6.3% (26) worked continuously for 15-25 hours (Table 1).

**Table 1:** Distribution of Sociodemographic risk factors ^*Modified Kuppuswamy scale ^ Data are presented as frequency (n) and percentage (%). The socioeconomic class was categorized based on the Modified Kuppuswamy Scale [21].

Variables	Categories	Frequency (n)	Percentage (%)
Age (in years)	18-40	59	14.2
	41-60	251	60.5
	>60	105	25.3
Gender	Male	300	72.3
	Female	115	27.7
Level of education	Primary school and below	272	65.5
	Secondary school and above	143	34.5
Type of occupation	Fishing	272	65.5
	Fish peeling	72	17.3
	Fish vending	46	11.1
	Fishnet repair	25	6
Socio-economic class^*^	Lower middle class	8	1.9
	Upper lower class	169	40.7
	Lower class	238	57.3
Duration of occupation (in hours)	0-20	178	42.9
	21-40	189	45.5
	>40	48	11.6
Continuous hours of work in the occupation (in hours)	1 – 14	389	93.7
	15- 25	26	6.3

Among the 415 participants, 28.7% (119) reported current tobacco use, while a higher proportion of 58.1% (241) had a history of past tobacco use. Current alcohol use was prevalent among 56.6% (235) of the participants, whereas 58.3% (242) had a history of past alcohol consumption. 88.9% (369) of participants reported having an inadequate fruit and vegetable intake, with only 11.1% (46) following an adequate fruit and vegetable intake. 33.5% (139) of the participants were classified as physically inactive, while 66.5% (276) were physically active. Sleep inadequacy was a significant concern, with 90.1% (374) of the participants reporting inadequate sleep, whereas only 9.9% (41) had adequate sleep duration. The prevalence of NCDs was notably high in the study population. Hypertension was reported in 57.8% (240) of participants, while 62.2% (258) of them were overweight or obese. Additionally, abdominal obesity was present in 93% (386) of participants, indicating a high burden of metabolic risk factors (Table 2).

**Table 2:** Distribution of behavioral risk factors and non-communicable diseases (NCDs) Data are presented as frequency (n) and percentage (%). Behavioral risk factors include tobacco and alcohol use, dietary habits, physical inactivity, and sleep duration. The prevalence of hypertension, overweight/obesity, and abdominal obesity among participants is also shown.

Variables	Categories	Frequency (n)	Percentage (%)
Current tobacco use	Yes	119	28.7
	No	296	71.3
Past tobacco use	Yes	241	58.1
	No	174	41.9
Current alcohol use	Yes	235	56.6
	No	180	43.4
Past alcohol use	Yes	242	58.3
	No	173	41.7
Dietary habits	Inadequate fruit and vegetable intake	369	88.9
	Adequate fruit and vegetable intake	46	11.1
Physical activity	Physically inactive	139	33.5
	Physically active	276	66.5
Sleep duration	Inadequate Sleep	374	90.1
	Adequate sleep	41	9.9
Distribution of NCDs			
Hypertension	Yes	240	57.8
	No	175	42.2
Overweight and obesity	Yes	258	62.2
	No	157	37.8
Abdominal obesity	Yes	386	93
	No	29	7

Based on the results obtained from the chi-square test, the prevalence of hypertension significantly increased with age, with only 22.0% (13/59) of individuals aged 18-40 years being hypertensive, compared to 62.9% (158/251) in the 41-60 age group and 65.7% (69/105) in those above 60 years (p < 0.001). Education level was significantly associated with hypertension, with a higher prevalence among those with primary education or below [63.2% (172/272)] compared to those with secondary education or above [47.6% (68/143)] (p = 0.002). Work duration was also a significant risk factor, as individuals working 21-40 hours per week had a higher risk of hypertension [64.6% (122/189)] compared to those working 0-20 hours [50.0% (89/178)] (p = 0.017). Additionally, income level showed a significant association, with hypertension more common among those in the upper lower class [72.2% (122/169)] compared to the lower class [47.1% (112/238)] (p < 0.001).

Overweight and obesity were significantly associated with age, with the lowest prevalence in the 41-60 age group [55.8% (140/251)], compared to 69.5% (41/59) in the 18-40 age group and 73.3% (77/105) in those above 60 years (p = 0.004). Males had a significantly higher risk [68.0% (204/300)] compared to females [47.0% (54/115)] (p < 0.001). Males also had a significantly higher risk of abdominal obesity [100% (300/300)] compared to females [74.8% (86/115)] (p < 0.001). Education level was significantly associated with abdominal obesity, with those having secondary education or above [97.2% (139/143)] exhibiting a higher risk compared to those with primary education or below [90.8% (247/272)] (p = 0.015). Occupation played a crucial role, as those engaged in fishing [99.6% (271/272)] and fish net repair [100% (25/25)] had the highest risk, while fish vendors had the lowest [73.9% (34/46)] (p < 0.001). The prevalence of abdominal obesity increased with longer working hours, with 97.9% (47/48) of individuals working more than 40 hours per week being affected (p = 0.031) (Table 3).

**Table 3:** Association of socio-demographic risk factors with non-communicable diseases (NCDs) Data are presented as frequency (n) and percentage (%). The chi-square (χ²) test was used to calculate the p-values. The p-values indicate the statistical significance of associations, with p < 0.05 considered significant. Significant values are marked with *.

Variables	Categories	Hypertension		p-value	Test statistic (χ²)	Overweight and obese		p-value	Test statistic (χ²)	Abdominal obesity		p-value	Test statistic (χ²)
		Yes n (%)	No n (%)			Yes n (%)	No n (%)			Yes n (%)	No n (%)		
Age (in years)	18 - 40	13(22.0)	46(78.0)	<0.001*	36.373	41(69.5)	18(30.5)	0.004^*^	11.270	57(96.6)	2(3.4)	0.451	1.593
	41 - 60	158(62.9)	93(37.1)			140(55.8)	111(44.2)			233(92.8)	18(7.2)		
	> 60	69(65.7)	36(34.3)			77(73.3)	28(26.7)			96(91.4)	9(8.6)		
Gender	Male	166(55.3)	134(44.7)	0.096	2.770	204(68.0)	96(32.0)	<0.001*	15.652	300(100)	0(0.0)	<0.001*	81.336
	Female	74(64.3)	41(35.7)			54(47.0)	61(53.0)			86(74.8)	29(25.2)		
Level of education	Primary school and below	172(63.2)	100(36.8)	0.002^*^	9.453	167(61.4)	105(38.6)	0.655	0.200	247(90.8)	25(9.2)	0.015^*^	5.895
	Secondary school and above	68(47.6)	75(52.4)			91(63.6)	52(36.4)			139(97.2)	4(2.8)		
Type of occupation	Fishing	144(52.9)	128(47.1)	0.017^*^	10.184	183(67.3)	89(32.7)	0.003^*^	13.623	271(99.6)	1(0.4)	<0.001^*^	71.745
	Fish peeling	48(66.7)	24(33.3)			32(44.4)	40(55.6)			56(77.8)	16(22.2)		
	Fish vending	28(60.9)	18(39.1)			26(56.5)	20(43.5)			34(73.9)	12(26.1)		
	Fish net repair	20(80.0)	5(20.0)			17(68.0)	8(32.0)			25(100.0)	0(0.0)		
Duration of occupation (in hours)	0-20	89(50.0)	89(50.0)	0.017^*^	8.107	105(59.0)	73(41.0)	0.069	5.356	159(89.3)	19(10.7)	0.031^*^	6.939
	21-40	122(64.6)	67(35.4)			116(61.4)	73(38.6)			180(95.2)	9(4.8)		
	>40	29(60.4)	19(39.6)			37(77.1)	11(22.9)			47(97.9)	1(2.1)		
Hours spent in occupation (in hours)	1 – 14 hours	232(59.6)	157(40.4)	0.004^*^	8.330	239(61.4)	150(38.6)	0.236	1.403	360(92.5)	29(7.5)	0.149	2.084
	15 – 25 hours	8(30.8)	18(69.2)			19(73.1)	7(26.9)			26(100.0)	0(0.0)		
Monthly income	Lower middle class	6(75.0)	2(25.0)	<0.001^*^	26.579	6(75.0)	2(25.0)	0.066	5.441	8(100.0)	0(0.0)	0.685	0.756
	Upper lower class	122(72.2)	47(27.8)			94(55.6)	75(44.4)			156(92.3)	13(7.7)		
	Lower class	112(47.1)	126(52.9)			158(66.4)	80(33.6)			222(93.3)	16(6.7)		

Current tobacco users had a significantly higher risk of hypertension [65.5% (78/119)] compared to non-users [54.7% (162/296)] (p = 0.044). Similarly, overweight and obesity were significantly more common among current tobacco users [70.6% (84/119)] compared to non-users [58.8% (174/296)] (p = 0.025). All current tobacco users had abdominal obesity [100% (119/119)], compared to 90.2% (267/296) of non-users, showing a strong association (p < 0.001). Past tobacco use was also significantly associated with overweight/obesity [69.3% (167/241) vs. 52.3% (91/174) (p < 0.001). Current alcohol use was associated with a significantly higher risk of overweight/obesity [68.5% (161/235) vs. 53.9% (97/180)] (p = 0.002) and abdominal obesity [100% (235/235) vs. 83.9% (151/180)] (p < 0.001). Past alcohol use showed similar patterns, with significantly higher overweight/obesity [69.0% (167/242) vs. 52.6% (91/173)] (p = 0.001) and abdominal obesity [100% (242/242) vs. 83.2% (144/173)] (p < 0.001). Abdominal obesity was present in all physically active individuals [100% (276/276)], while physically inactive individuals had a lower prevalence [79.1% (110/139)] (p < 0.001). Sleep duration was significantly associated with hypertension, with inadequate sleepers [61.0% (228/374)] showing a higher prevalence compared to those with adequate sleep [29.3% (12/41)] (p < 0.001) (Table 4).

**Table 4:** Association of Behavioral risk factors with non-communicable diseases (NCDs) Data are presented as frequency (n) and percentage (%). The chi-square (χ²) test was used to calculate the p-values. The p-values indicate the statistical significance of associations, with p < 0.05 considered significant. Significant values are marked with *. Behavioral risk factors analyzed include tobacco and alcohol use, dietary habits, physical activity, and sleep duration in relation to hypertension, overweight/obesity, and abdominal obesity.

Variable	Category	Hypertension		p value	Test statistic (χ²)	Overweight and obesity		p value	Test statistic (χ²)	Abdominal obesity		p value	Test statistic (χ²)
		Yes n (%)	No n (%)			Yes n (%)	No n (%)			Yes n (%)	No n (%)		
Current tobacco users	Yes	78(65.5)	41(34.5)	0.044^*^	4.072	84(70.6)	35(29.4)	0.025^*^	5.029	119(100.0)	0(0.0)	<0.001^*^	12.535
	No	162(54.7)	134(45.3)			174(58.8)	122(41.2)			267(90.2 )	29(9.8)		
Past tobacco users	Yes	149(61.8)	92(38.2)	0.052	3.761	167(69.3)	74(30.7)	<0.001^*^	12.410	240(99.6)	1(0.4)	<0.001^*^	38.208
	No	91(52.3)	83(47.7)			91(52.3)	83(47.7)			146(83.9)	28(16.1)		
Current alcohol users	Yes	141(60.0)	94(40.0)	0.307	1.045	161(68.5)	74(31.5)	0.002^*^	9.265	235(100.0)	0(0.0)	<0.001^*^	40.706
	No	99(55.0)	81(45.0)			97(53.9)	83(46.1)			151(83.9)	29(16.1)		
Past alcohol users	Yes	145(59.9)	97(40.1)	0.309	1.036	167(69.0)	75(31.0)	0.001^*^	11.547	242(100.0)	0(0.0)	<0.001^*^	43.614
	No	95(54.9)	78(45.1)			91(52.6)	82(47.4)			144(83.2)	29(16.8)		
Diet habits	Adequate fruit and vegetable intake	21(45.7)	25(54.3)	0.076	3.147	31(67.4)	15(32.6)	0.439	0.600	44(95.7)	2(4.3)	0.456	0.555
	Inadequate fruit and vegetable intake	219(59.3)	150(40.7)			227(61.5)	142(38.5)			342(92.7)	27(7.3)		
Physical activity	Physically inactive	91(65.5)	48(34.5)	0.025^*^	4.998	71(51.1)	68(48.9)	0.001^*^	10.928	110(79.1)	29(20.9)	<0.001^*^	61.909
	Physically active	149(54.0)	127(46.0)			187(67.8)	89(32.2)			276(100.0)	0(0.0)		
Sleep duration	Inadequate sleep	228(61.0)	146(39.0)	<0.001^*^	15.220	232(62.0)	142(38.0)	0.862	0.030	348(93.0)	26(7.0)	0.931	0.008
	Adequate sleep	12(29.3)	29(70.7)			26(63.4)	15(36.6)			38(92.7)	3(7.3)		

Multivariable logistic regression analysis shows that past smoking was a strong predictor of abdominal obesity, with past smokers having significantly higher odds of abdominal obesity (adjusted odds ratio (AOR) = 42.99, 95% confidence interval (CI): 5.777-319.918, p < 0.001) compared to non-smokers. However, educational level did not show a statistically significant association with abdominal obesity (AOR = 0.352, 95% CI: 0.116-1.066, p = 0.065).

Gender was identified to be a significant risk factor of overweight and obesity, with males having 2.4 times higher odds compared to females (AOR = 2.40, 95% CI: 1.547-3.724, p < 0.001). Age did not show a significant association, with participants aged 41-60 years (AOR = 0.71, 95% CI: 0.380-1.346, p = 0.298) and those above 60 years (AOR = 1.53, 95% CI: 0.734-3.199, p = 0.255) having similar odds to the 18-40 age group. Income was also not significantly associated with overweight and obesity.

Hypertension was significantly associated with age, past smoking, and sleep duration. Participants aged 41-60 years had 3.29 times higher odds of hypertension compared to those aged 18-40 years (AOR = 3.286, 95% CI: 1.587-6.806, p = 0.001), while those above 60 years had similar odds (AOR = 3.216, 95% CI: 1.427-7.249, p = 0.005). Past smokers had 2.75 times higher odds of developing hypertension than non-smokers (AOR = 2.753, 95% CI: 1.392-5.444, p = 0.004). Sleep duration was another significant predictor, with those experiencing inadequate sleep having 3.39 times higher odds of hypertension than those with adequate sleep (AOR = 3.389, 95% CI: 1.565-7.340, p = 0.002). Occupation was also associated with hypertension, as individuals engaged in fishing had lower odds compared to those in fish net repair (AOR = 0.313, 95% CI: 0.105-0.928, p = 0.036). However, diet did not show a significant association with hypertension (AOR = 1.910, 95% CI: 0.977-3.733, p = 0.058) (Table 5).

**Table 5:** Factors associated with abdominal obesity, overweight and obesity and hypertension - results of multivariable logistic regression analysis Data are presented as adjusted odds ratio (AOR) with 95% CI. A multivariable logistic regression model was used to identify significant predictors. The p-values indicate the statistical significance of associations, with p < 0.05 considered significant. Significant values are marked with *.

Factors associated with abdominal obesity					
Variables	Category	AOR	95% C.I for AOR		p-value
			Lower	Upper	
Past smoker	Yes	42.99	5.777	319.918	<0.001^*^
	No	1			
Education	Primary school and below	0.352	0.116	1.066	0.065
	Secondary school and above	1			
Factors associated with Overweight and obesity					
Age	18-40	1			
	41-60	0.71	0.380	1.346	0.298
	>60	1.53	0.734	3.199	0.255
Gender	Male	2.40	1.547	3.724	<0.001^*^
	Female	1			
Income	Lower middle class	1			
	Upper lower class	0.594	0.113	3.122	0.539
	Lower class	0.963	0.183	5.064	0.965
Factors associated with Hypertension					
Age	18-40	1			
	41-60	3.286	1.587	6.806	0.001^*^
	>60	3.216	1.427	7.249	0.005^*^
Occupation	Fishing	0.313	0.105	0.928	0.036^*^
	Fish peeling	1.003	0.270	3.719	0.997
	Fish vending	0.846	0.222	3.223	0.807
	Fish net repair	1			
Income	Lower middle class	1			
	Upper lower class	0.709	0.131	3.835	0.690
	Lower class	0.286	0.053	1.525	0.143
Past smoker	Yes	2.753	1.392	5.444	0.004^*^
	No	1			
Diet	Inadequate fruit and vegetable intake	1.910	0.977	3.733	0.058
	Adequate fruit and vegetable intake	1			
Sleep duration	Inadequate sleep	3.389	1.565	7.340	0.002^*^
	Adequate sleep	1			

Awareness and treatment behavior of NCDs (hypertension, diabetes, high cholesterol)

Individuals with hypertension, diabetes, and high cholesterol were aware of their conditions, but only a subset received treatment. Among the 57.8% (240) diagnosed with hypertension, 55.4% (230) were aware of their condition, but only 45.1% (187) were receiving treatment. For diabetes, 27.5% (114) of participants were aware of their diagnosis, yet only 23.1% (96) were undergoing treatment. Similarly, 23.6% (98) were aware of having high cholesterol, but only 19.8% (82) were receiving treatment.

## Discussion

There has been limited research on NCDs in the fishing community, particularly among fisherwomen in Kerala. Previous studies have primarily focused on fishermen, with minimal attention given to fisherwomen. In the fishing community, older age groups are traditionally engaged in fishing activities, relying solely on fishing for their livelihood. However, it is noteworthy that many are now pursuing at least a high school education. Our findings show that the majority of participants had completed primary and secondary education, and most were involved in fishing occupations. Fisherwomen primarily engage in fish selling, fishing in backwaters, head-load fish vending, fish peeling, and shell collection. As these activities depend entirely on fishing, participants' income levels varied, but most were from middle- or lower-middle-class backgrounds. The duration of their work was also considerable, with many working for over 21 years and some spending 15 to 25 hours continuously in their occupations. Fishing activities can vary in duration due to weather conditions and the quantity of fish caught. While fishing in backwaters requires less time and effort, the prolonged work hours often affect sleep and dietary habits, increasing the risk of NCDs. This study provides critical insights into the associations of behavioral, lifestyle, and sociodemographic factors with abdominal obesity, overweight/obesity, and hypertension in the fishing community, particularly among fisherwomen [22].

Abdominal obesity

Past smoking emerged as a strong predictor of abdominal obesity, with past smokers exhibiting 43 times higher odds compared to non-smokers. These results align with previous studies demonstrating that smoking contributes to visceral fat accumulation through mechanisms such as insulin resistance and hormonal imbalances. Similar results were observed in studies conducted in coastal Tamil Nadu, where smoking was significantly associated with abdominal obesity, underscoring the need for region-specific smoking cessation programs [23]. In a study on Indian fishermen [24], a high prevalence of abdominal obesity was reported despite their physically demanding occupations, underscoring that environmental and behavioral factors play a crucial role in shaping obesity patterns in this community. The higher prevalence of abdominal obesity compared to overweight and general obesity may be attributed to the dietary habits and physical activity patterns of the fishing community. Many fishermen consume a high-carbohydrate diet, leading to central fat accumulation despite not being classified as overweight by BMI. Additionally, occupational factors such as irregular meal timings and high alcohol consumption may contribute to increased visceral fat deposition. This can be supported with a population study done on the Iranian population [25].

Overweight and obesity

Age, gender, and socioeconomic status were significant predictors of overweight/obesity. Males were over twice as likely to be overweight or obese compared to females. This finding is consistent with national studies in India, such as those in Tamil Nadu, and international research from the United States and Japan, which attribute higher obesity rates in men to factors such as alcohol consumption and dietary behaviors. Additionally, individuals from the lower socioeconomic class had significantly higher odds of overweight/obesity compared to those from the upper-lower class. The Indian Council of Medical Research-India Diabetes (ICMR-INDIAB-3) study [26] found that socioeconomic factors significantly contributed to obesity in urban and rural areas of Tamil Nadu, highlighting the interplay between economic constraints and dietary habits, which mirrors the findings in our study. Studies from low- and middle-income countries, such as Brazil and South Africa, further support these findings, demonstrating that socioeconomic disparities exacerbate obesity risk [27]. A study from Denmark showed similar results to our study, with 37% of fishermen aged 45 to 64 being overweight or obese [28].

Hypertension

The prevalence of hypertension in this study (57.8%, 240 out of 415 participants) was higher than that reported in the general rural population of Kerala (19.82%), indicating a higher burden of NCDs in the fishing community [29]. Advancing age was a strong predictor, with individuals aged over 60 years having three times higher odds of hypertension compared to those aged 18-40 years. Rahman SN et al. [30] highlighted the prevalence of hypertension in rural fishermen communities in coastal regions of Bengal, noting that lifestyle factors such as high salt intake and inadequate physical activity contributed significantly to hypertension, reinforcing our findings on the role of diet and physical activity. Bansal et al. [31] discussed hypertension in rural populations across India, finding a strong association with dietary habits, sedentary behavior, and aging, which corresponds with the predictors identified in our study. Behavioral factors, including diet and sleep, were also significant predictors of hypertension. Participants with inadequate diets had higher odds of hypertension. Similarly, inadequate sleep was associated with nearly threefold higher odds of hypertension. Past smoking was identified as a significant predictor of hypertension, with past smokers having twice the odds of developing the condition. Similar results supporting our study were found in a study conducted in Chennai, India, where 180 fishermen had a hypertension prevalence of 38.9%. Significant associations were identified between hypertension, age, and years of fishing experience [32].

Strengths

Our study successfully identified the risk factor profile for NCDs among the fishing community, a population that has been relatively underrepresented in NCD research in Kerala. While previous studies on NCDs in Kerala have primarily focused on coastal areas, our study specifically targeted individuals engaged in fishery-related activities, providing a more nuanced understanding of the health risks in this community. Moreover, while most studies have concentrated on male fishermen, our research also included fisherwomen, which offers a more comprehensive picture of the health risks faced by this population. The use of cluster random sampling for participant selection enhances the robustness of our findings and strengthens the generalizability of our results to the fishing community in Ernakulam district. Additionally, our study assessed the awareness and behavioral perceptions of NCDs, which could inform the development of tailored awareness programs aimed at improving health outcomes within the community.

Limitations

A major limitation of this study was the inability to collect biochemical measurements due to the COVID-19 pandemic, which resulted in significant disruptions during the data collection period. This limited our ability to obtain objective measures of critical NCD risk factors such as blood glucose and lipid levels, which are essential for a more accurate assessment of participants' health status. Additionally, the availability of fishermen for participation was challenging, as many of them were frequently out at sea for extended periods. As a result, some participants self-reported conditions such as diabetes and high cholesterol, which may have introduced bias due to recall errors or misreporting. The presence of partners or parents during interviews could have influenced participants' responses, especially regarding sensitive topics like substance use, introducing potential social desirability bias. Furthermore, our study did not examine multimorbidity within the fishing community, an area that warrants further investigation to provide a more comprehensive understanding of the health burden in this population. Future studies exploring the prevalence and impact of multimorbidity would contribute valuable insights for developing targeted health interventions in the fishing community.

## Conclusions

The study reveals a high prevalence of NCD risk factors among the fishing community, emphasizing the significant burden of hypertension, obesity, and inadequate sleep, particularly among older individuals. Despite a majority being aware of their health conditions, treatment uptake remains low, highlighting gaps in healthcare access and awareness. Behavioral and sociodemographic factors such as tobacco use, alcohol consumption, inadequate fruit and vegetable intake, and physical inactivity were significantly associated with NCDs, underscoring the urgent need for targeted interventions. This research, which uniquely includes both fishermen and fisherwomen, advances understanding of the NCD burden in marginalized occupational groups and emphasizes the importance of tailored, community-based public health strategies to mitigate these risks and improve overall health outcomes in the fishing community. Addressing the high prevalence of NCDs in the fishing community requires targeted awareness programs on lifestyle changes, improved access to affordable screening and treatment, and community-based health promotion initiatives. Policies should focus on improving living conditions and integrating health education into occupational settings. Future efforts should prioritize inclusive strategies that address gender-specific needs and explore the impact of multimorbidity for sustainable health interventions.

## Appendices

Appendix 1

**Table 6:** Questionnaire Adapted from the WHO STEP-wise Approach to Noncommunicable Disease Risk Factor Surveillance (STEPS) instrument [18], with modifications to suit the study population and location

DEMOGRAPHIC INFORMATION					CODE
Sex	Male				C1
	Female				
Age					C2
In total, how many years have you spent at school and in full-time study (excluding pre-school)?					C3
What is the highest level of education you have completed?	No formal schooling				C4
	Less than primary school				
	Primary school completed				
	Secondary school completed				
	High school completed				
	College/University completed				
	Postgraduate degree				
	Refused				
What is your marital status?	Never married				C5
	Currently married				
	Separated				
	Divorced				
	Widowed				
	Cohabitating				
	Refused				
Type of labor					C6
Duration in fishing occupation					C7
Frequency of fishing per month/week					C8
Number of hours spent in the sea					C9
How many people older than 18 years, including yourself, live in your household?	Number of people:				C10
Taking the past year, can you tell me what the average Earnings of the household have been?	Per week:				C11
	OR per month				
	OR per year				
	Refused 88				
STEP 1 BEHAVIOURAL MEASUREMENTS					
TOBACCO USE					
Do you currently smoke any tobacco products, such as cigarettes, or cigars?	Yes				T1
	No				
	if no go to T7				
Do you currently smoke tobacco products daily?	Yes				T2
	No				
How old were you when you first started smoking?	Age (years)				T3
	Don’t know				
Do you remember how long ago it was?	In Years				T4
	OR in Months				
	OR in Weeks				
On average, how many times a day/week do you use ….		Daily	Weekly		T5
	Snuff, by mouth				
	Snuff, by the nose				
	Chewing tobacco				
	Betel, quid				
	Other				
	Other (please specify):				
During the past 12 months, have you tried to stop smoking?	Yes				T6
	No				
During any visit to a doctor or other health worker in the past 12 months, were you advised to quit smoking tobacco?	Yes				T7
	No				
	No visit during the past 12 months				
In the past, did you ever smoke any tobacco products?	Yes				T8
	No				
In the past, did you ever smoke daily?	Yes				T9
	No				
How old were you when you stopped smoking?	Age (years)				T10
	Don’t Know				
	………….Years ago				
	………….Months ago				
	………….Weeks ago				
How long ago did you stop smoking?	Years ago/Months ago/Weeks ago				T11
Do you use any smokeless tobacco products such as [snuff, chewing tobacco, betel]?	Yes				T12
	No				
Do you currently use smokeless tobacco products daily?	Yes				T13
	No				
On average, how many times a day/week do you use		Daily		Weekly	T14
	Snuff, by mouth				
	Snuff, by the nose				
	Chewing tobacco				
	Betel, quid				
	Other				
	Other (please specify):				
In the past, did you ever use smokeless tobacco products such as [snuff, chewing tobacco, or betel]?	Yes				T15
	No				
	If no go to T17				
In the past did you ever use smokeless tobacco products such as [ snuff, chewing tobacco, or betel] daily?	Yes				T16
	No				
During the past 30 days, did someone smoke in your home?	Yes				T17
	No				
During the past 30 days, did someone smoke in closed areas in your workplace (in the building in a work area or a specific office)?	Yes				T18
	No				
	Don't work in a closed area				
Alcohol Consumption					
Have you ever consumed any alcohol such as beer, wine, spirits or [add other local examples]?	Yes				A1
	No				
	if no go to A12				
Have you consumed any alcohol within the past 12 months?	Yes				A2
	No				
Have you stopped drinking due to health reasons such as negative impact on your health or on the advice of your doctor or other health worker?	Yes				A3
	No				
During the past 12 months, how frequently have you had at least one standard alcoholic drink?	Daily				A4
	5-6 days per week				
	3-4 days per week				
	1-2 days per week				
	1-3 days per month				
	Less than once a month				
	never				
Have you consumed any alcohol within the past 30 days?	Yes				A5
	No				
In the last 30 days, counting all the alcoholic beverages together, what is the highest number of standard drinks you drank in a single session?	Largest number :				A6
	Don‘t know				
During the past 30 days, how many times did you have six or more standard drinks in a single drinking occasion?	Number of times:				A7
	Don‘t know				
During each of the past 7 days, how many standard drinks did you have each day?	Monday :				A8
	Tuesday :				
	Wednesday :				
	Thursday :				
	Friday ;				
	Saturday ;				
	Sunday :				
	Don’t know				
During the past 12 months, how often have you found that you were not able to stop drinking once you had started?	Daily or almost daily				A9
	Weekly				
	Monthly				
	Less than monthly				
	Never				
During the past 12 months, how often have you failed to do what was normally expected from you because of drinking?	Daily or almost daily				A10
	Weekly				
	Monthly				
	Less than monthly				
	Never				
During the past 12 months, how often have you needed a first drink in the morning to get yourself going after a heavy drinking session?	Daily or almost daily				A11
	Weekly				
	Monthly				
	Less than monthly				
	Never				
During the past 12 months, have you had family problems or problems with your partner due to someone else’s drinking?	Yes, more than monthly				A12
	Yes, monthly				
	Yes, several times but less than				
	Monthly				
	Yes, once or twice				
	No				
DIET					
In a typical week, how many days do you eat fruit?	Number of days:				D1
How many servings of fruit do you eat on one of those days?	Number of servings:				D2
In a typical week, how many days do you eat vegetables?	Number of days:				D3
How many servings of vegetables do you eat on one of those days?	Number of servings:				D4
DIETARY SALT					
How often do you add salt or a salty sauce such as soy sauce to your food right before you eat it or as you are eating it?	Always				D5
	Often				
	Sometimes				
	Rarely				
	Never				
	Don't know				
How often is salt, salty seasoning, or a salty sauce added in cooking or preparing foods in your household?	Always				D6
	Often				
	Sometimes				
	Rarely				
	Never				
	Don't know				
How often do you eat processed food high in salt? By processed food high in salt, I mean foods that have been altered from their natural state, such as packaged salty snacks, canned salty food including pickles and preserves, salty food prepared at a fast-food restaurant, cheese, bacon, and processed meat	Always				D7
	Often				
	Sometimes				
	Rarely				
	Never				
	Don't know				
How much salt or salty sauce do you think you consume?	Far too much				D8
	Too much				
	Just the right amount				
	Too little				
	Far too little				
	Don't know				
How important to you is lowering the salt in your diet	Very important				D9
	Somewhat important				
	Not at all important				
	Don't know				
Do you think that too much salt or salty sauce in your diet could cause a health problem?	Yes				D10
	No				
	Don't know				
Do you do any of the following regularly to control your salt intake?					
Limit consumption of processed foods	Yes				D11a
	No				
Look at the salt or sodium content on food labels	Yes				D11b
	No				
Buy low salt/sodium alternatives	Yes				D11c
	No				
Use spices other than salt when cooking	Yes				D11d
	No				
Avoid eating foods prepared outside of a home	Yes				D11e
	No				
Do other things specifically to control your salt intake	Yes				D11f
	No				
Other (please specify)					D11 other
PHYSICAL ACTIVITY					
Does your work involve vigorous-intensity activity that causes large increases in breathing or heart rate like [carrying or lifting heavy loads, digging, or construction work] for at least 10 minutes continuously?	Yes				P1
	No				
	If no go to P4				
In a typical week, how many days do you do vigorous-intensity activities as part of your work?	Number of days:				P2
How much time do you spend doing vigorous-intensity activities at work on a typical day?	Hours: minutes				P3
Does your work involve moderate-intensity activity, that causes small increases in breathing or heart rate such as brisk walking [or carrying light loads] for at least 10 minutes continuously?	Yes				P4
	No				
	If no go to P7				
In a typical week, how many days do you do moderate-intensity Activities as part of your work?	Number of days:				P5
How much time do you spend doing moderate-intensity activities at work on a typical day?	Hours: minutes				P6
Travel to and from places					
Do you walk or use a bicycle (pedal cycle) for at least 10 minutes continuously to get to and from places?	Yes				P7
	No				
	If no go to P10				
In a typical week, on how many days do you walk or bicycle for at least 10 minutes continuously to get to and from places?	Number of days:				P8
How much time do you spend walking or bicycling for travel on a typical day?	Hours: minutes				P9
Recreational activities					
Do you do any vigorous-intensity sports, fitness, or recreational (leisure) activities that cause large increases in breathing or heart rate like [running or football] for at least 10 minutes continuously?	Yes				P10
	No				
	If no go to P13				
In a typical week, how many days do you do vigorous intensity sports, fitness, or recreational (leisure) activities?	Number of days:				P11
How much time do you spend doing vigorous-intensity sports, fitness, or recreational activities on a typical day?	Hours: minutes				P12
Do you do any moderate-intensity sports, fitness, or recreational (leisure) activities that cause a small increase in breathing or heart rate such as brisk walking, [cycling, swimming, volleyball] for at least 10 minutes continuously?	Yes				P13
	No				
	If no go to P16				
In a typical week, how many days do you do moderate-intensity sports, fitness, or recreational (leisure) activities?	Number of days				P14
How much time do you spend doing moderate-intensity sports, fitness, or recreational (leisure) activities on a typical day?	Hours: minutes				P15
Sedentary behavior					
How much time do you usually spend sitting or reclining on a typical day?	Hours: minutes				P16
Hours of sleep:					S1
History of Raised Blood Pressure					
Have you ever had your blood pressure measured by a doctor or other health worker?	Yes				H1
	No				
	If no, go to H6				
Have you ever been told by a doctor or other health worker that you have raised blood pressure or hypertension?	Yes				H2a
	No				
	If no, go to H6				
Were you first told in the past 12 months?	Yes				H2b
	No				
In the past two weeks, have you taken any drugs (medication) for raised blood pressure prescribed by a doctor or other health worker?	Yes				H3
	No				
Have you ever seen a traditional healer for raised blood pressure or hypertension?	Yes				H4
	No				
Are you currently taking any herbal or traditional remedies for your raised blood pressure?	Yes				H5
	No				
History of Diabetes					
Have you ever had your blood sugar measured by a doctor or other health worker?	Yes				H6
	No				
	if no go to H12				
Have you ever been told by a doctor or other health worker that you have raised blood sugar or diabetes?	Yes				H7a
	No				
Were you first told in the past 12 months?	Yes				H7b
	No				
In the past two weeks, have you taken any drugs (medication) for diabetes prescribed by a doctor or other health worker?	Yes				H8
	No				
Are you currently taking insulin for diabetes prescribed by a doctor or other health worker?	Yes				H9
	No				
Have you ever seen a traditional healer for diabetes or raised blood sugar?	Yes				H10
	No				
Are you currently taking any herbal or traditional remedies for your diabetes?	Yes				H11
	No				
History of Raised Total Cholesterol					
Have you ever had your cholesterol (fat levels in your blood) measured by a doctor or other health worker?	Yes				H12
	No				
	If no go to H17				
Have you ever been told by a doctor or other health worker that you have raised cholesterol?	Yes				H13a
	No				
Were you first told in the past 12 months?	Yes				H13b
	No				
In the past two weeks, have you taken any oral treatment (medication) for raised total cholesterol prescribed by a doctor or other health worker?	Yes				H14
	No				
Have you ever seen a traditional healer for raised cholesterol?	Yes				H15
	No				
Are you currently taking any herbal or traditional remedies for your raised cholesterol?	Yes				H16
	No				
History of Cardiovascular Diseases					
Have you ever had a heart attack or chest pain from heart disease (angina) or a stroke (cerebrovascular accident or Incident)?	Yes				H17
	No				
Are you currently taking aspirin regularly to prevent or treat heart disease?	Yes				H18
	No				
Are you currently taking statins (Lovastatin/Simvastatin/Atorvastatin or any other statin) regularly to prevent or treat heart disease?	Yes				H19
	No				
CORE (for women only): Cervical Cancer Screening					
The next question asks about cervical cancer prevention. Screening tests for cervical cancer prevention can be done in different ways, including Visual Inspection with Acetic Acid/vinegar (VIA), pap smear, and Human Papillomavirus (HPV) test. VIA is an inspection of the surface of the uterine cervix after acetic acid (or vinegar) has been applied to it. For both pap smear and HPV test, a doctor or nurse uses a swab to wipe from inside your vagina, take a sample and send it to a laboratory. It is even possible that you were given the swab yourself and asked to swab the inside of your vagina. The laboratory checks for abnormal cell changes if a pap smear is done, and for the HP virus if an HPV test is done					
Have you ever had a screening test for cervical cancer, using any of the methods described above?	Yes				W1
	No				
	Don‘t know				
Lifestyle Advice					
During the past 12 months, have you visited a doctor or other health worker?	Yes				L1
	No				
During any of your visits to a doctor or other health worker in the past 12 months, were you advised to do any of the following?					L2
Quit using tobacco or don‘t start	Yes				L2a
	No				
Reduce salt in your diet	Yes				L2b
	No				
Eat at least five servings of fruit and/or vegetables each day	Yes				L2c
	No				
Reduce fat in your diet	Yes				L2d
	No				
Start or do more physical activity	Yes				L2e
	No				
Maintain a healthy body weight or lose weight	Yes				L2f
	No				
Reduce sugary beverages in your diet	Yes				L2g
	No				
PHYSICAL MEASUREMENTS					
BLOOD PRESSURE					
Cuff size used	Small				M1
	Medium				
	Large				
Reading 1	Systolic (mmHg):				M2
	Diastolic (mmHg):				
Reading 2	Systolic (mmHg):				
	Diastolic (mmHg):				
Average:					
During the past two weeks, have you been treated for raised blood pressure with drugs (medication) prescribed by a doctor or other health worker?	Yes				M3
	No				
HEIGHT AND WEIGHT					
Height	in Centimetres (cm)				M4
Weight	in Kilograms (kg)				M5
BMI					M6
Waist circumference: in cm					M7
Hip circumference: in cm					M8
Heart rate: in beats per minute - Reading 1:					M9
